# Coupling ITO3dE model and GIS for spatiotemporal evolution analysis of agricultural non-point source pollution risks in Chongqing in China

**DOI:** 10.1038/s41598-021-84075-2

**Published:** 2021-02-25

**Authors:** Kang-wen Zhu, Zhi-min Yang, Lei Huang, Yu-cheng Chen, Sheng Zhang, Hai-ling Xiong, Sheng Wu, Bo Lei

**Affiliations:** 1 College of Resources and Environment, Southwest University, Chongqing, 400716 China; 2 Chongqing Academy of Ecology and Environmental Sciences, Chongqing, 401147 China; 3 College of Computer & Information Science, Southwest University, Chongqing, 400716 China

**Keywords:** Agroecology, Ecological modelling, Ecosystem ecology, Environmental sciences

## Abstract

To determine the risk state distribution, risk level, and risk evolution situation of agricultural non-point source pollution (AGNPS), we built an ‘Input-Translate-Output’ three-dimensional evaluation (ITO3dE) model that involved 12 factors under the support of GIS and analyzed the spatiotemporal evolution characteristics of AGNPS risks from 2005 to 2015 in Chongqing by using GIS space matrix, kernel density analysis, and Getis-Ord Gi* analysis. Land use changes during the 10 years had a certain influence on the AGNPS risk. The risk values in 2005, 2010, and 2015 were in the ranges of 0.40–2.28, 0.41–2.57, and 0.41–2.28, respectively, with the main distribution regions being the western regions of Chongqing (Dazu, Jiangjin, etc.) and other counties such as Dianjiang, Liangping, Kaizhou, Wanzhou, and Zhongxian. The spatiotemporal transition matrix could well exhibit the risk transition situation, and the risks generally showed no changes over time. The proportions of ‘no-risk no-change’, ‘low-risk no-change’, and ‘medium-risk no-change’ were 10.86%, 33.42%, and 17.25%, respectively, accounting for 61.53% of the coverage area of Chongqing. The proportions of risk increase, risk decline, and risk fluctuation were 13.45%, 17.66%, and 7.36%, respectively. Kernel density analysis was suitable to explore high-risk gathering areas. The peak values of kernel density in the three periods were around 1110, suggesting that the maximum gathering degree of medium-risk pattern spots basically showed no changes, but the spatial positions of high-risk gathering areas somehow changed. Getis-Ord Gi* analysis was suitable to explore the relationships between hot and cold spots. Counties with high pollution risks were Yongchuan, Shapingba, Dianjiang, Liangping, northwestern Fengdu, and Zhongxian, while counties with low risks were Chengkou, Wuxi, Wushan, Pengshui, and Rongchang. High-value hot spot zones gradually dominated in the northeast of Chongqing, while low-value cold spot zones gradually dominated in the Midwest. Our results provide a scientific base for the development of strategies to prevent and control AGNPS in Chongqing.

## Introduction

Agricultural non-point source pollution (AGNPS) refers to water pollution caused by nitrogen and phosphorus, pesticides, and other contaminants through farmland water runoff and percolation^1^. In China, in the prevention and control of water pollution, point sources have mainly been taken into consideration, while non-point sources are largely being ignored^2^. However, along with the effective control of point source pollution, AGNPS has gradually attracted more and more attention. Currently, the premise of effectively solving the problem of AGNPS lies in the accurate evaluation of the risk state distribution, the risk level, and the risk evolution situation of AGNPS. In particular, the integration of numerous technologies and methods, such as models for the calculation of AGNPS^3^, the universal soil loss equation (USLE)^4^, and GIS technology^5^, has greatly promoted the research on AGNPS in China^6^.

According to the comparison of the results of the two pollution source surveys released by China in 2020, the total amount of water pollutant emissions decreased significantly in the past ten years (2007–2017)^7^. The proportion of agricultural sources in chemical oxygen demand, nitrogen pollutants and total phosphorus emissions increased from 43.71%, 41.88%, 67.27% to 49.77%, 40.73%, 67.22% respectively. It shows that the contribution of AGNPS to water pollution is very high^8^. The city of Chongqing is characterized by a hilly and mountainous landform, a shattered topography, a high proportion of rural areas in the urban and rural dual structure, a hot and rainy season, and a high and concentrated precipitation, which result in a high potential threat, a wide coverage area, and a high driving energy of AGNPS in Chongqing^9,10^. The application amount of chemical fertilizer, pesticide and agricultural film in Chongqing is increasing constantly. In 2014, the application level of chemical fertilizer, pesticide and agricultural film reached 411 kg ha, 9.5 kg ha and 81.9 kg ha respectively, which was far higher than the international standard upper limit. In addition, due to the influence of topography and climate on soil erosion, the total amount of soil erosion is 146 million tons per year. In addition, there are other problems such as high multiple cropping index of agricultural land in Chongqing^11^. Chongqing is located in the center of the Three Gorges Reservoir area and represents the connection point of the "Belt and Road" and the Yangtze River economic belt. Hence, Chongqing has an important status in the national regional development pattern and is an important ecological barrier of the upstream of the Yangtze River; in this sense, strict water management strategies are indispensable. To ensure the ecological security of the Yangtze River basin, the issue of AGNPS needs to be resolved. In view of this, it is of great importance to understand the spatiotemporal evolution situation of AGNPS risks in districts and counties of Chongqing. To sum up, there are several problems to be solved in research fields and risk assessment of AGNPS. (1) Research on the spatiotemporal evolution of AGNPS in Chongqing or other large regions are scarce. (2) In terms of risk evaluation, on an international level, the most commonly used technologies and methods include the export coefficient approach^12^, the pollution index method (phosphorus index method)^13^, the multi-factor index evaluation method^14^, and the model evaluation method of non-point source pollution (NPS)^15^, and there are few studies on the integration of agronomy and geography. (3) There are many studies on input and output dimensions in AGNPS risk assessment^16^, but few studies consider translate dimension.

This research adopts the method of combining multi factor index and GIS spatial analysis, which was more advanced in AGNPS risk analysis at present, and could also enrich the research results in this field. And Wu built N and P load calculation models in the Three Gorges Reservoir area by combined with RUSLE equation and GIS technology, and also confirmed the advantages of the combination of the two methods^17^. Therefore, in our study, based on the comprehensive analysis of previous studies and regional characteristics of Chongqing^6,10^, and combining the advantages of GIS technology and the multi factor index comprehensive evaluation method. An ‘Input-Translate-Output’ three-dimensions evaluation (ITO3dE) model was built to evaluate the risks of AGNPS. There were 12 factors involved in the ITO3dE model, including input dimension (fertilizer use intensity, pesticide use intensity, livestock intensity), translate dimension (erosion caused by rainfall, slope length and gradient, soil erodibility, sloping field, distance from water area), and output dimension (water quality, water capacity, water network density, degree of paddy field retention), selected by expert consultation and system analytic hierarchy processes. On this basis, the weights of these factors were determined through multiple assignment by combination with the Delphi method^18^.

The spatial overlay analysis function of GIS was adopted to conduct a comprehensive evaluation and combined with the results of related research on safe reference values of various factors. The spatiotemporal variation of the AGNPS risks in Chongqing from 2005 to 2015 was analyzed by using space matrix analysis, kernel density analysis, and Getis-Ord Gi*. To reflect the characteristics of each factor in the large-scale spatialization process, it was assumed that the factors fertilizer use intensity and pesticide use intensity were evenly dispersed in the farmland area, that the livestock intensity factor was evenly dispersed in the suitable breeding area, and that the paddy field retention factor was evenly dispersed in the paddy field area.

Therefore, the spatiotemporal changes of the AGNPS risk on a large scale are studied in this research. AGNPS was mainly caused by the application of chemical fertilizer, pesticides and livestock farming, which were prevalent in the world. The research method takes into account the three dimensions of input, translate, and output. The research results have the advantages of a high visibility of risk results, identifiable risk levels, and analyzable risk changes. In addition, the study combines regional land use change and links some factors with a certain type of land use. Compared with other studies, it can better reflect the spatial differences and could therefore solve the problems of a wide coverage area of AGNPS and of the difficulty of accurately identifying high-risk areas^19,20^.

## Research methods

### Data sources

Data types were mainly divided into panel data, remote sensing data, and statistical data, at a resolution of 30 m. The remote sensing data included land use data for 2005, 2010 and 2015, soil type data, digital elevation model (DEM) terrain data, slope data, river data and zones suitable for breeding. The land use data were derived from China's Eco-environmental Remote Sensing Assessment Project with a resolution of 30 m^21^. The DEM data were obtained from the resource and environment data cloud platform with a resolution of 30 m (http://www.resdc.cn/). The slope data were calculated by DEM data in GIS software, while the river data were extracted from high-resolution remote sensing images. Data on zones suitable for breeding were obtained from the delimited projects about the three livestock and poultry breeding zones (i.e., non-breeding zones, breeding-restricted zones, and zones suitable for breeding), issued by the Chongqing Agriculture and Rural Affairs Committee.

The statistical data included fertilizer use, pesticide use, crop planting area, livestock and poultry breeding data, COD, TN, and TP data, and water generation modulus data. Data on fertilizer use, pesticide use, and crop planting area were derived from the Chongqing data system (http://www.cqdata.gov.cn/). Livestock and poultry breeding data were obtained from the Chongqing Agriculture and Rural Affairs Committee (http://nyncw.cq.gov.cn/), while data on the levels of COD, TN, and TP were provided by the Chongqing Bureau of Ecology and Environment (http://sthjj.cq.gov.cn/). Water generation modulus data were obtained from the Chongqing Water Resource Environment Bulletin (http://slj.cq.gov.cn/).

### Study area

**Figure 1:**
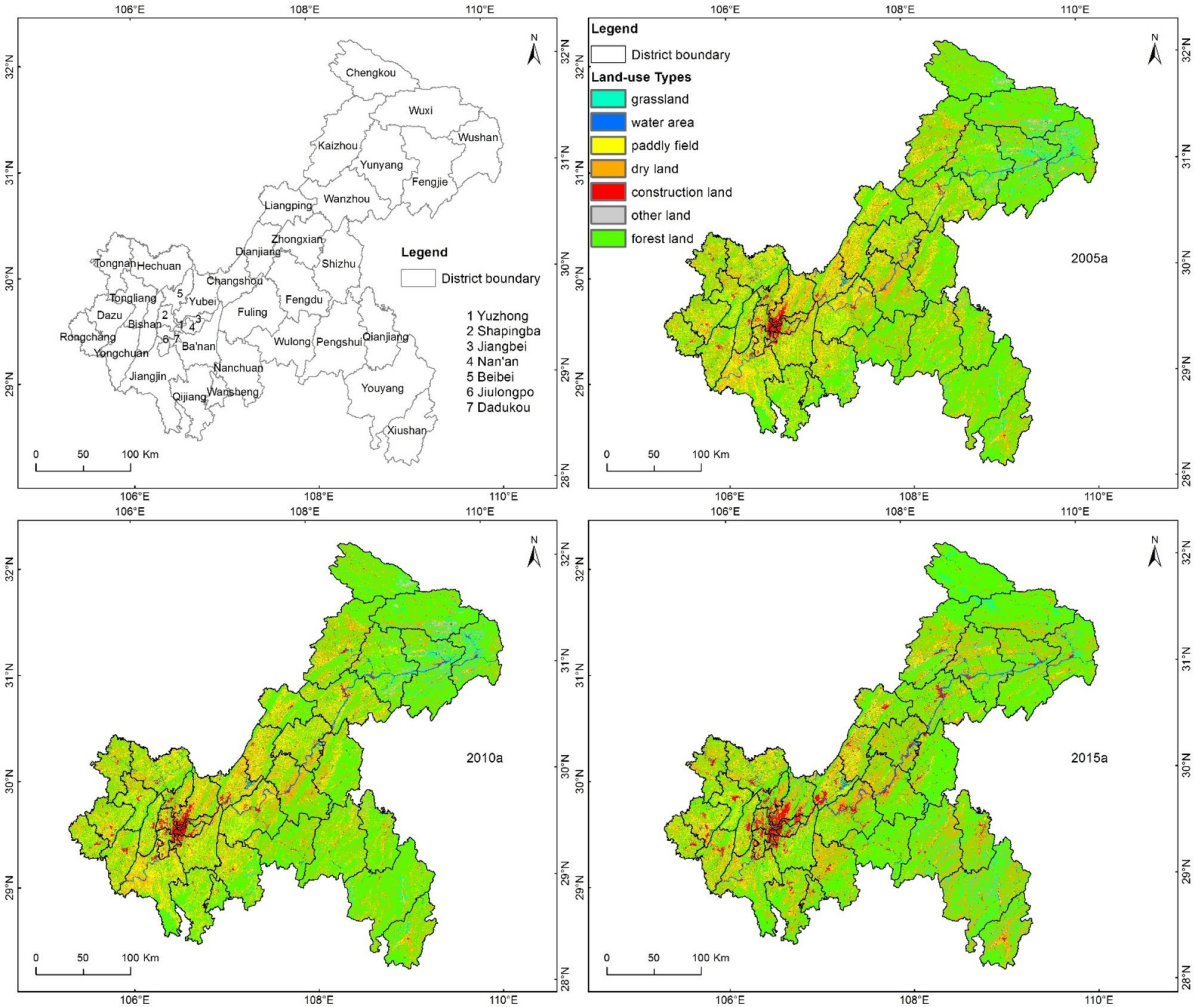
Land use map of Chongqing in 2005, 2010 and 2015.

Chongqing is located in southwestern China and is one of China's four municipalities directly under the central government. The current fertilizer application level in Chongqing is 411 kg ha, while the internationally recognized safe upper limit is 225 kg ha. More seriously, the fertilizer use rate is only about 35%. Pesticide use intensity is 9.5 kg ha, with a use rate of only 30%. Livestock and poultry stocks are currently increasing significantly. In addition, the hilly and mountainous areas in Chongqing account for 94% of the total area, while the cultivated land area with a slope greater than 25 degrees accounts for 16% of the total cultivated land area, which is 11 percentage points higher than the national average level. Rainfall in Chongqing is large and concentrated, and the area subjected to soil erosion accounts for 48.6% of the total area^11^. Based on the schematic illustration of the land use types in Chongqing from 2005 to 2015, the artificial surface area displays significant external diffusion in the main urban area of Chongqing, the western region of Chongqing, Changshou County, Kaizhou County, and Wanzhou County. In contrast, the area of paddy fields shows a significantly decreasing trend, while the area of dry land is relatively stable; farmland is mainly distributed in the western region as well as in Wanzhou County, Kaizhou County, Liangping County, and Dianjiang County in the northeastern region. According to the data analysis results, the water area accounts for 1.59–1.96% of the coverage area of Chongqing. The area of paddy fields decreased from 14.8% in 2005 and 2010 to 7.64% in 2015, while the area of dry land is relatively stable at 25%. The artificial surface area has increased from 1.42 to 3.18%, while the proportion of forested land showed a steady increase (Fig. 1). Overall, the farmland area of Chongqing showed a significantly decreasing trend, with a reduction from 39.35% to 33.03%. Farmland is the main source of AGNPS. To sum up, the actual regional conditions of land use types, topography, climate, fertilizer and pesticide application lead to the aggravation of AGNPS in Chongqing.

### Research methods

**Figure 2:**
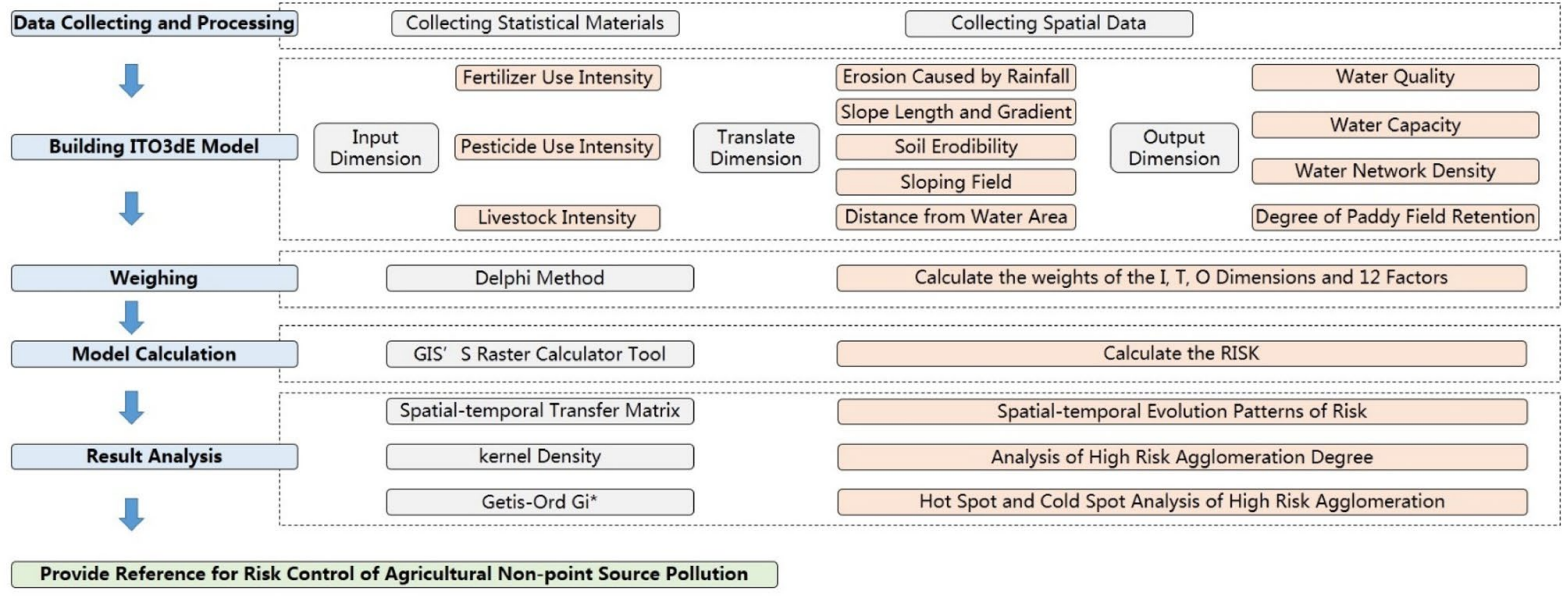
The research technology frame.

The risk assessment of AGNPS in Chongqing was carried out by constructing the ITO3dE model, and the specific content was analyzed by using GIS technology. The overall framework of the study was as follows (Fig. 2).

#### ITO3dE model building

The ITO3dE model was built from three dimensions of “Input-Translate-Output” (Table 1). Here, the I dimension (*A*_1_) includes fertilizer use intensity (*I*_1_)^22^, pesticide use intensity (*I*_2_)^23^, and livestock intensity (*I*_3_)^24^, the T dimension (*A*_2_) includes erosion caused by rainfall (*I*_4_), slope length and gradient (*I*_5_), soil erodibility (*I*_6_), sloping field (*I*_7_), and distance from water area (*I*_8_), and the O dimension (*A*_3_) includes water quality (*I*_9_)^25^, water capacity (*I*_10_)^26^, water network density (*I*_11_)^27^, and degree of paddy field retention (*I*_12_).

**Table 1:** Calculation methods and reference values of the indices in the ITO3dE model.

Dimension	Index	Calculation method	Reference value	Source of reference value	Direction of index
Input dimension (*A1*)	*I*_1_	Chemical fertilizer use intensity per unit of farmland area	250 kg ha	Building indicators of national ecological town/ county/ city/ province	Positive
	*I*_2_	Pesticide use intensity per unit of farmland area	2.5 kg ha	Internationally recognized secure usage level	Positive
	*I*_3_	Calculating the livestock pollution load ratio, the deficiency ratio of environmentally protective facilities for large-scale breeding, and the ratio of large-scale breeding to small-scale breeding by the Nemerrow index^23^ environmental quality comprehensive evaluation method			Positive
Translate dimension (*A2*)	*I*_4_	Simplified calculation method of R value, proposed by Zhou et al.^58^	100 J cm ha h	Provisional regulations of ecological function zoning	Positive
	*I*_5_	Calculating the index by a 7 × 7 window through ArcGIS neighborhood statistics	50	Provisional regulations of ecological function zoning	Positive
	*I*_6_	Calculating the K values (soil erodibility) of various land use types based on previous related research and the second overall soil examination results of Chongqing^59^	Grading standard in provisional regulations of ecological function zoning		Positive
	*I*_7_	Calculating the index according to the grading standard of soil erodibility classification by combining with farmland data and slope data	Field slope of 15°	Grading standard of soil erodibility classification	Positive
	*I*_8_	Calculating the index by the spatial distance analysis function in GIS, based on the data of three-level above rivers and lakes in the study area	1500 m	Existing literature and document ^60^	Negative
Output dimension (*A3*)	*I*_9_	Calculating the index by computing pollution indices of COD, NH_3_-N, and TP, using the Nemerow index			Positive
	*I*_10_	The ratio of overall runoff generation modulus to regional runoff generation modulus in the study area	61.95	Long-term average value during 2005–2015 in Chongqing	Negative
	*I*_11_	Calculating the index according to the lake and reservoir density and the river density according to the ecological environment status evaluation^61^	0.025 km^2 km^-2 and 0.404 km km^-2 are the reference values of the lake and reservoir density and the river density respectively	Long-term average value during 2005–2015 in Chongqing	Negative
	*I*_12_	Proportion of paddy field area in farmland area	0.3339	Long-term average value during 2005–2015 in Chongqing	Negative

The computational formula of positive indices is as follows:

1 $${I}_{i}={C}_{i}/{E}_{i}$$

The computational formula of negative indices is as follows:

2 $${I}_{i}={E}_{i}/{C}_{i}$$

In the above equations, *I*_*i*_ denotes the calculation result of a certain index of *i*, *C*_*i*_ is the true value of the *i* index, and *E*_*i*_ is the reference value. The calculation method, reference value and source of reference value involved in ITO3dE model calculation were described in Table 1. The calculation results for each index can be classified into five grades of no risk, low risk, medium risk, high risk, and extremely high risk, corresponding to the values of ≤ 0.7, 0.7–1.0, 1.0–3.0, 3.0–5.0, and ≥ 5.0. This grading standard refers to the relevant technical planning of China's agricultural sector^28^, and the grading standard of this study was written based on Chongqing local standard "Specification for evaluation risk of AGNPS in Chongqing".

#### Delphi method

The weights of the evaluation indices of AGNPS risks were determined by the Delphi method^18^. We distributed 120 questionnaires to the leaders of related departments engaged in agricultural and rural work, environmental work, and water conservancy programs, as well as to experts at colleges, universities, and research institutes, and to technical personnel at the grass-roots level; in total, we received 115 valid questionnaires. After the first round of weights assignment, we returned the calculation results to the experts for adjusting the index weights, representing the second round of weights assignment. According to the evaluation opinions of the experts, we again revised the evaluation indices and conducted the third round of weights assignment to obtain the final weights results. Based on these, we could obtain the multifactor comprehensive evaluation relational expressions as follows:

3 $$A=0.430{A}_{1}+{0.231A}_{2}+{0.339A}_{3}$$

4 $${A}_{1}={0.547I}_{1}+{0.339I}_{2}+{0.114I}_{3}$$

5 $${A}_{2}={0.290{I}_{4}+0.098I}_{5}+{0.182I}_{6}+{0.267I}_{7}+0.163{I}_{8}$$

6 $${A}_{3}={0.347I}_{9}+{0.293I}_{10}+{0.153I}_{11}+0.207{I}_{12}$$

In the above equations, the letter *A* represents the comprehensive risk result of AGNPS, while the other letters have the same meanings as in Table 1, and indicating the calculation results of each index.

#### Spatiotemporal transition matrix of risk index

Transition matrices are widely used in analyzing the spatiotemporal variations and can clearly exhibit the variations of risk indices at different periods^29^. In this study, we assigned the five risk grades to the values of 1, 2, 3, 4, and 5, respectively, and subsequently employed the following formula to analyze the risk status:

7 $${{B}_{M}=B}_{2005}\times 100+{B}_{2010}\times 10+{B}_{2015}$$

where *B*_*2005*_, *B*_*2010*_, *B*_*2015*_ is the operation layer and *B*_*M*_ is the result layer. In the calculation results, "123" represents the risk transition process in a certain region from no risk in 2005 to low risk in 2010 and to medium risk in 2015; the remaining results can be interpreted in the same manner.

#### Kernel density analysis of risk index

Kernel density is mainly used to analyze the spatial concentration of an event and is widely used in the distribution of buildings, schools, and criminal activities^30^. The kernel density can reflect the concentration degree and agglomeration location of AGNPS above a high risk level. Kernel density estimation is a spatial analysis method based on nonparametric testing^30,31^. The basic idea of kernel density estimation for certain elements is to assume that there always exists an element intensity at an arbitrarily position within a particular region. The density intensity of geographical elements in a specific location can then be estimated by measuring the number of elements per unit area. By determining the element density at different locations and the spatial differences, the relative concentration degree of the spatial distribution of elements can be depicted, and the hotspot distribution regions can be identified. Subsequently, using the kernel density analysis tool in the ArcGIS software, we can analyze the grids at risk grades of high risk and extremely high risk and explore the extreme point position of AGNPS in our study area, Chongqing.

#### Getis-Ord Gi* analysis

The Getis-Ord Gi* analysis is widely used in crime analysis, epidemiology and economic geography, and is used to identify spatial gathering of high values (hot spots) and low values (cold spots) with statistical significance^32^. In Getis-Ord Gi* analysis, the z score, p values, and confidence intervals (Gi_Bin) are employed to create a new output class for each element in the input element class. Here, the z score and p values can help to judge whether the null hypothesis can be rejected, while the Gi_Bin field is used to identify statistically significant hot and cold spots. The elements in the confidence interval of [+ 3, − 3] have a statistical significance with a confidence level of 99%, while those in the confidence interval of [+ 2, − 2] have a statistical significance with a confidence level of 95%, and those in the confidence interval of [+ 1, − 1] have a statistical significance with a confidence level of 90%; when the element gathering of Gi_Bin field is 0, there is no statistical significance.

### Statement

We confirm that all experimental protocols were approved by College of Resources and Environment of Southwest University, all methods were carried out in accordance with relevant guidelines and regulations of questionnaires, and confirming that informed consent was obtained from all subjects or, if subjects are under 18, from a parent and/or legal guardian.

## Results

### Results of risk assessment by the ITO3dE model

**Figure 3:**
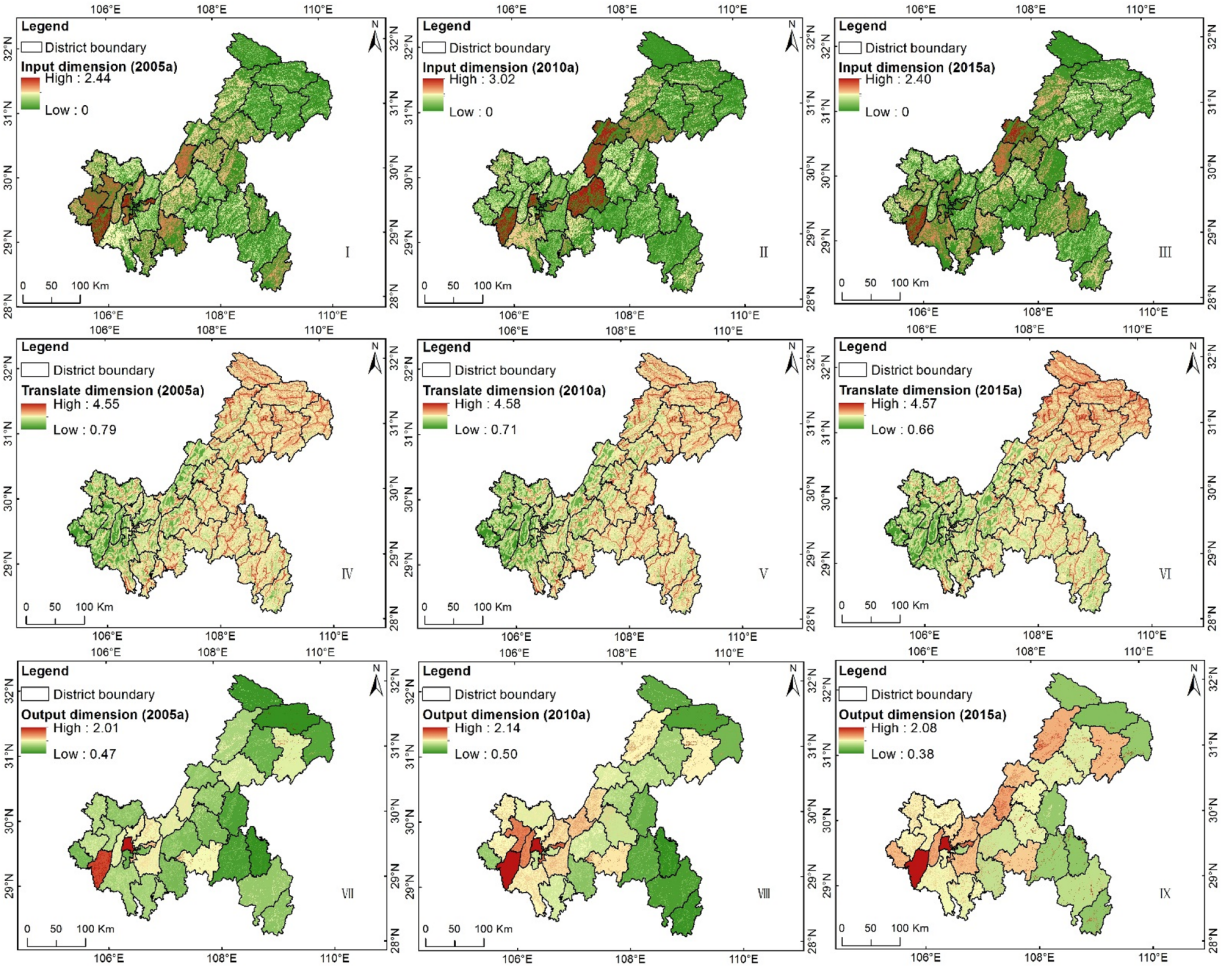
Result distribution map of I, T, and O dimensions of Chongqing in 2005, 2010, 2015.

The results in the I dimension show that, overall, the distribution was high in the west and low in the northeast and the southeast in all three periods (Fig. 3, I, II, III; Table 2), and this tallies with the topography of Chongqing. The northwestern and central regions of Chongqing are mainly hilly and slightly mountainous, while the southeastern and northeastern regions represent the Dabashan Mountain system and the Daloushan Mountain system, respectively. Thus, farmland in Chongqing is mainly distributed in the western regions as well as in regions with extensive flat areas, such as Dianjiang and Liangping. Some regions in Dianjiang, Yongchuan, Dazu, Shapingba, Wansheng, and Jiangbei show relatively high risks, but the risk level is still medium. Hence, it can be concluded that the risk level in the I dimension during 2005–2015 is, overall, not high. Considering there are too many single-factor graphs, we omitted these graphs, but provide the following description: Among the three single factors, *I*_*1*_ has the highest value, and *I*_*1*_ and *I*_*2*_ both present a first increasing and then decreasing trend (the maximum values of I_1_ in 2005, 2010, and 2015 were 3.38, 4.08, and 2.78, respectively, and those of *I*_*2*_ in 2005, 2010, and 2015 were 2.71, 3.37, and 2.48, respectively). For the *I*_*1*_ results, the risk levels of the regions with higher levels in 2005, such as Yongchuan, Fuling, and Liangping, showed a certain decrease in 2015, but the risk levels of some regions such as Pengshui, Qianjiang, and Xiushan showed an increasing trend. The risk grade of *I*_*2*_ was relatively lower than that of *I*_*1*_, but overall, the spatiotemporal variation trend was consistent with that of *I*_*1*_, except for the increasing trend of the risk level of Qianjiang. Basically, the risk grade of *I*_*3*_ was zero; only the risk level of Bishan was in the medium risk status, while those of Hechuan and Fengdu were low.

**Table 2:** Statistical results of I, T, and O dimensions in 2005–2015.

Dimension	Year	Range of value	Districts and counties with high value
Input	2005	0–2.44	Yongchuan, Shapingba, Jiangbei, Jiulongpo, Dadukou, Dazu, Tongliang, Diangjiang, Nanchuan
	2010	0–3.02	Yongchuan, Dadukou, Shapingba,Fuling, Liangping, Dianjiang
	2015	0–2.40	Yongchuan, Liangping, Dadukou, Nan’An, Wansheng, Jiangjin, Jiulongpo, Shapingba, Dianjiang
Translate	2005	0.79–4.55	Chengkou, Wuxi, Wushan, Fengjie, Yunyang, Kaizhou, Wanzhou, Shizhu, Zhongxian, Fuling, Fengdou, Wulong, Pengshui, Qianqiang, Youyang, Xiushan
	2010	0.71–4.58	
	2015	0.66–4.57	
Output	2005	0.47–2.01	Yongchuan, Shapingba, South of Wulong,
	2010	0.50–2.14	Yongchuan, Tongliang, Bishan, Shapingba, Dadukou, Jiangbei
	2015	0.38–2.08	Yongchuan, Shapingba, Rongchang, Bishan, Dianjiang, Jiangbei

Spatially, the results in the T dimension presented, overall, an opposite distribution pattern when compared to the I dimension, that is, with low levels in the western regions and high levels in the northeastern and southeastern regions (Fig. 3, IV, V, VI; Table 2). The annual differences in the T dimension data are mainly determined by the variations in the factors *I*_*4*_ and *I*_*7*_, which showed relatively higher risk levels in all three periods. The values of *I*_*4*_ in the years 2005, 2010, and 2015 were 1.42–5.78, 0.84–6.12, and 0.14–6.93, respectively, while those of *I*_*7*_ were 0, 0–5.38, and 0–5.06, respectively. Because Chongqing is a typical mountainous city with purple soil^33^, high-risk and extremely high-risk regions, *I*_*5*_ and *I*_*6*_, are widely distributed across the city. In addition, due to the introduction of the factor *I*_*8*_, the water areas had a higher risk level, which is consistent with the actual situation of AGNPS.

The results in the O dimension showed a smaller interannual variation, with a low overall risk level (Fig. 3, VII, VIII, IX; Table 2). The O dimension levels were mainly affected by the spatial changes in the paddy field area. As mentioned above, during the 10 years, the area of paddy fields in Chongqing was nearly reduced by half, which led to the decrease in the spatial distribution of *I*_*12*_ and an increased risk in counties such as Kaizhou, Fengjie, Liangping, and Changshou. Spatially, Yongchuan, Shapingba, Bishan, Dianjiang, Changshou, and Kaizhou showed higher risk levels, and the risk levels of Kaizhou, Fengjie, Wanzhou, Liangping, and Changshou showed a significantly increasing trend. The high risk values of *I*_*9*_ were mainly distributed in Yongchuan, Shapingba, Jiangbei, Changshou, Dianjiang, and Liangping, with Shapingba showing the highest value of 3.75, while Chengkou, Wushan, Fengjie, Shizhu, and Xiushan had lower values. The high risk values of *I*_*10*_ were mainly distributed in the western regions and were below the medium risk levels. The risk values in 2010 were higher than those in 2005 or 2015, but did not surpass 3.0, and the high values were mainly distributed in the western regions as well as in Dianjiang, Wanzhou, and Liangping. The risk values of *I*_*11*_ were all below 3.0, and the highest value of 2.78 was found for Fengjie; higher values were mainly distributed in the northeastern and southeastern counties. The high risk values of I_12_ were mainly distributed in the northeastern and southeastern counties, which mostly have only small areas of paddy fields.

**Figure 4:**
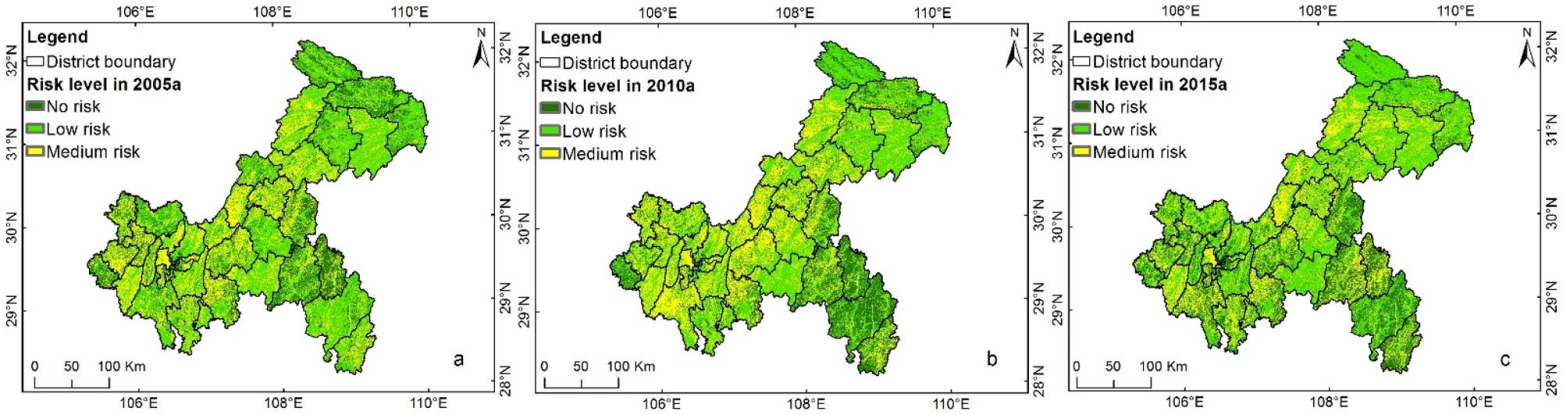
Spatiotemporal distribution graph of the evaluation results of agricultural NPSP risks in Chongqing during 2005–2015: (**a**) 2005; (**b**) 2010; (**c**) 2015.

Figure 4 shows the data on AGNPS risks during 2005–2015 in Chongqing. The risk distribution trends in 2005, 2010, and 2015 were basically consistent and in the ranges of 0.40–2.28, 0.41–2.57, and 0.41–2.28, respectively. The maximum risk values were all below 3.0 for the three periods. Regions with medium levels were mostly distributed in the western regions of Chongqing (Dazu, Jiangjin, etc.) as well as in the counties Dianjiang, Liangping, Kaizhou, Wanzhou, and Zhongxian. Larger spatial differences were observed among different counties or different parts of a certain county; for example, the middle flatland part and the mountain systems at the two sides in Liangping or the northwestern and southeastern parts in Shizhu.

### Spatiotemporal change results of risk by transition matrix analysis

**Figure 5:**
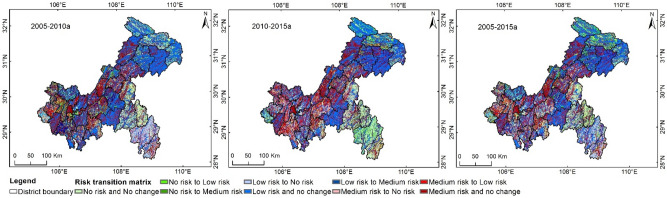
Spatiotemporal transition situation of agricultural NPSP risks in Chongqing during 2005–2015.

By assigning no risk, low risk, and medium risk levels with 1, 2, and 3, respectively, in GIS, we can obtain the spatiotemporal transition matrix according to the formula of the transition matrix. Figure 5 shows the spatiotemporal transition situation of the AGNPS risk evaluation in Chongqing. Basically, high levels show no changes, and the proportions of ‘no-risk no-change’, ‘low-risk no-change’, and ‘medium-risk no-change’ situations were 10.86%, 33.42%, and 17.25%, respectively, accounting for 61.53% of the total area of Chongqing. Among these, the ‘no-risk no-change’ situation was mainly distributed in Rongchang, the east of Nanchuan, Shizhu, Pengshui, and Qianjiang; the ‘low-risk no-change’ situation was widely distributed in Wulong, the southeast of Fengdu, the south of Nanchuan, and the northeastern counties of Chongqing, while the ‘medium-risk no-change’ situation was mainly distributed in Shapingba, Yongchuan, Dianjiang, the north of Nanchuan, and Kaizhou.

During 2005–2015, the proportions of risk increase, risk decline, and risk fluctuation were 13.45%, 17.66%, and 7.36%, respectively. Risk increases mainly occurred in central Jiangjin, central Fengdu, Pengshui, Qianjiang, the midwest of Yunyang, central Liangping, Wuxi, Wushan, and Chengkou, while risk declines were mainly observed for the main urban area of Chongqing, northern Tongliang, Dazu, Youyang, and Xiushan. Risk fluctuation was concentrated in Jiangjin, Bishan, Fuling, and Youyang.

### Results of risk concentration degree by Kernel density analysis

**Figure 6:**
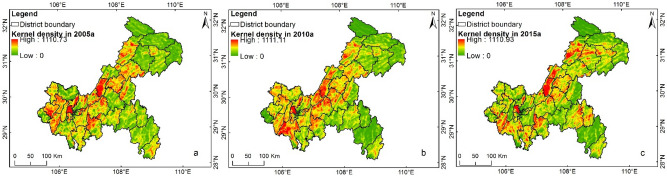
Kernel density graphs of medium-risk areas in Chongqing during 2005–2015: (**a**) 2005; (**b**) 2010; (**c**) 2015.

Figure 6 shows the kernel density analysis results of the medium-risk regions. As seen in the figures, the peak values of the kernel density at these three periods were all around 1,110, suggesting that the maximum gathering degree of medium-risk pattern spots basically showed no changes. The spatial distribution of kernel density at these three periods showed a consistent trend, but the distribution differences at different periods were significant. In 2005, medium-risk regions were mainly concentrated in Shapingba, southern Dazu, central Yongchuan, eastern Beibei, Dianjiang, central Kaizhou, northwestern Shizhu, northern Nanchuan, central Wanzhou, southwestern Zhongxian, and southeastern Xiushan, while in 2010, such regions mainly occurred in Shapingba, eastern Jiangjin, southeastern Beibei, northern Nanchuan, northeastern Changshou, Dianjiang, northern Fuling, northern Fengdu, northeastern Shizhu, northeastern Liangping, central Kaizhou, Wanzhou, northeastern Pengshui, and eastern Xiushan. In 2015, medium-risk regions were mainly concentrated in Shapingba, Yongchuan, central Jiangjin, northwestern Nanchuan, northeastern Beibei, Dianjiang, Liangping, the junction of Fuling and Fengdu, central Kaizhou, northern Yunyang, eastern Pengshui, southeastern Qianjiang, and central Xiushan.

To further explore the distribution of regions with the high-risk gathering zones (Table 3), we conducted a separate analysis on the regions with kernel density values higher than 1,000 (the kernel density values of these regions were divided into 10 grades with equal intervals, and the 10th grade had values from 1,000 to 1,110).

**Table 3:** Distribution of regions with high-risk gathering zones.

Years	The high-risk gathering zones
2005	The junction of Youting town and Longshui town in Dazu, the central part of Longshui town in Dazu, the east of Shapingba, the junction of Liuyin town and Sansheng town, as well as the junction of Liuyin town and Longshui town in Beibei, the southwest of Shiyan town in Changshou, the central part of Baijia town in Dianjiang, the junction of Yantai town and Chengxi town in Dianjiang, the junction of Gaofeng town and Gaoan town, as well as the junction of Gaofeng town and Gangjia town in Dianjiang, and the west of Nantuo town in Fuling
2010	The eastern part of Shapingba, the west of Jiangjin, the north of Youting town in Dazu, the south of Liuyin town and the junction of Jingguan town and Sansheng town in Beibei, the junction of Gelan town and Shiyan town in Changshou, Yihe town and Nantuo town in Fuling, the central part of Lidu subdistrict in Fuling, the central part of Baijia town in Dianjiang, the southeast of Chengxi town in Dianjiang, the junction of Gaofeng town and Gangjia town in Dianjiang, and Linjiang town in Kaizhou
2015	The eastern part of Shapingba, the north of Youting town in Dazu, the central part of Baijia town in Dianjiang, the southeast of Chengxi town in Dianjiang, the junction of Gaofeng town and Gangjia town in Dianjiang, and the western Linjiang town and central Wenquan town in Kaizhou

### Results of hot and cold spots by Getis-Ord Gi* analysis

**Figure 7:**
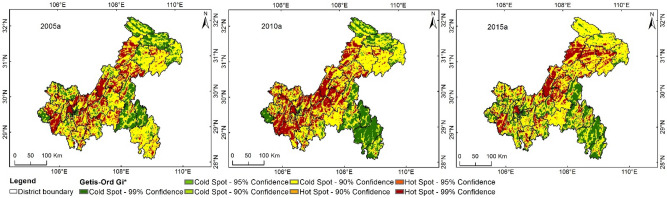
Getis-Ord Gi analysis results in Chongqing during 2005–2015.

Applying Getis-Ord Gi* analysis is helpful to clearly identify high-value hot spots (Hot Spot-99% Confidence) and low-value cold spots (Cold Spot-99% Confidence). Figure 7 shows the Getis-Ord Gi* analysis results; the overall variation trends of high-value hot spots and low-value cold spots were consistent in all periods, with significant distribution differences. The regions located in the high-value hot spot zones in all three periods were Yongchuan, Shapingba, Dianjiang, Liangping, northwestern Fengdu, and Zhongxian, while those located in the low-value cold spot zones were Chengkou, Wuxi, Wushan, Pengshui, and Rongchang. Throughout the 10 years, the high-value hot spot zones showed significant diffusion in Fengjie, Yunyang, Kaizhou, central Qianjiang, and northern Nanchuan, while the low-value cold spot zones showed significant diffusion in some parts of the midwestern counties such as central Fuling and southern Yubei. These high-value hot spots or low-value cold spots were mainly distributed in the above-mentioned regions and their surrounding areas and showed significant “gathering trends”. The spatiotemporal variation trend of the distribution of these high-value hot spots or low-value cold spots can reflect the variation tendencies of hot spots or cold spots in different regions. Over time, the high-value hot spot zones gradually migrated towards the northeastern counties of Chongqing, while the low-value cold spot zones in the midwestern counties presented an obvious diffusion trend. The low-value cold spot zones in the northeastern regions gradually decreased, while those in the southeastern regions tended to become more fragmented. These results indicate that the high-value hot spot zones gradually dominated the northeastern regions, while the low-value cold spot zones gradually dominated the midwestern regions.

## Discussion

### The I, T, and O dimensions could better comprehensively analyze the risk situation of AGNPS

There were many achievements in AGNPS risk research using different methods. For instance, Huang has analyzed the risk of NPS in Taihu Lake from multiple perspectives, and the method was only applicable to small-scale studies^35^. In addition, it focused on the distribution of pollution sources, and on waste disposal, but did not consider the process from pollution sources to water bodies. Blankenberg has analyzed the relationship between wetland and pesticide concentration in streams from the perspectives of pesticide spraying and changes in pesticide concentration in water bodies^36^. The research focused on the relationship between wetland and pesticide concentration in small watersheds, while the land use types, topographic conditions, rainfall, and other factors that affect concentration changes were not considered. This research therefore comprehensively considers input factors, translate factors and output factors.

The chemical fertilizers, pesticides, livestock and poultry factors selected in the I dimension of this study were recognized as the main sources of AGNPS^37–39^. In addition, the factors of the T dimension such as rainfall, slope length and gradient, soil erodibility, sloping field, and distance from water area have gradually increased in recent years. For instance, Zhang has analyzed the effects of rainfall intensity and slope on the loss of suspended solids and phosphorus in runoff^40^, which showed that the slope difference of sloping farmland was also an important factor affecting pollutant migration^41^, and the distance from the water reflected the difficulty degree of pollutants entering the water body^42^. The influence factors of the O dimension increased the water network density and the degree of paddy field retention factor compared with other studies. Most of the studies mainly focused on risk the analysis of AGNPS from water quality and water capacity^43,44^. In Wang et al.^34^ used the minimum cumulative resistance model to calculate the risk of ANSP of cultivated land in the Three Gorges Reservoir area, and believed that the topography, hydrology, soil and vegetation had a great impact on the risk of ANSP. In fact, high water network density and paddy field ratio could effectively reduce the pollution. In this study, the I, T, and O dimensions were considered comprehensively, and factors affecting AGNPS risk were added to the large-scale framework.

### Geographical methods were well applied in the field of agriculture

The use of geographic methods in this study makes the results of AGNPS risk analysis more intuitive and reflects the temporal and spatial changes of research results from different perspectives. The spatiotemporal transition matrix quantifies the variation in the different risk levels and can accurately identify the regions with increasing, decreasing, or unchanged risk levels. The spatiotemporal transition matrix analysis was one of the most widely used methods in geographic research and often used to study the change of the same spatial event over time. For instance, Shawul and Chakma have used this method to analysis the conversion of land use types in different periods^45^. The kernel density analysis could effectively identify the spatial agglomeration area of an event and was widely used in geographic, medical event analysis and case analysis^46^. The advantage of the Getis-Ord Gi* analysis is to judge the hot spots of high and low values at the same time and to analyze the evolution trend of hot spots of high and low values. This method was usually applied in studies that require the analysis of high-value and low-value changes simultaneously^47^. Zhang et al.^48^ used the hot spot analysis method in geography to analyze the spatial and temporal pattern of ANSP in Chongqing section of the Three Gorges Reservoir area, and achieved good results. In our research, we need to consider both high-risk and low-risk changes in AGNPS.

### Land variations influence AGNPS risks

The analysis of land use changes indicates that the land area in Chongqing has changed greatly during the 10-year-period from 2005 to 2015. In particular, the paddy field area was reduced by nearly 50%. Our observations are in agreement with previous findings^49^. The regions with declining farmland areas were mainly located in the urban areas of Chongqing and in the surroundings of the town, which goes hand in hand with the economic development and the urban expansion of Chongqing in recent years, reflecting the direct influences of urban development on AGNPS. This tallies with the decrease in chemical fertilizer use intensity and pesticide use intensity, as farmland reduction inevitably lead to a decrease in the application of these substances^50^, complying with the “one regulatory, two reduction, three basic” policy promoted by the Chinese government. A previous study has analyzed the relationship between the riparian forest buffer zone and pollution mitigation in Chesapeake bay watershed and found that the wide buffer zone could effectively reduce water pollution^51^. Other authors have analyzed the relationship between climate, land use change, and water quality in the Pike river watershed and found that land use changes had an impact on water quality^52^. The water quality factors in this study also reflected that the water quality in the southeast and northeast areas with high forest coverage was better, indicating that land use change had a great impact on water quality, especially on the cultivated land, which was closely related to the use of fertilizers and pesticides.

In this study, we analyzed the spatiotemporal variation in AGNPS in three dimensions of "Input-Transition-Output", with the conclusion that the risk levels in the Input and Output dimensions were lower, while that in the Translate dimension was higher. Our conclusion accords with the characteristics of Chongqing as a mountainous city; the widely distributed purple soil, the larger differences in slope gradient and slope length, and the widely distributed sloping fields are indeed conducive to the generation of AGNPS^53^. PENG et al.^54^ analysis of the driving factors of ANSP in the Three Gorges Reservoir Area (Chongqing Section) showed that land use type, agricultural production and living intensity have a greater impact on ANSP, which was consistent with our research conclusion. From 2005 to 2015, the counties with higher risk levels in all three periods were basically those national basic farmland demonstration counties (Jiangjin, Dazu, Tongnan, Tongliang, Liangping, Dianjiang), with higher risk levels in the Input and Output dimensions. These areas therefore require planting activities under the conditions of ensuring soil safety, water quality safety, and food security.

### Recommendations

#### Focus on risk increase area in the analysis of spatiotemporal transfer matrix

In 62% of our study area, the risk levels remained unchanged, indicating that the influencing factors of AGNPS in most regions of Chongqing are relatively stable. For about 14% of the study area, the risk levels showed an increasing trend, and these regions, especially food security zones such as Jiangjin and Liangping as well as important ecological shelter zones of the Three Gorges Reservoir areas such as Fengdu, Yunyang, Wuxi, and Wushan, need to be considered in management programs. AGNPS in these areas might seriously threaten the ecological security of the Yangtze River economic belt. Regional managers should control the use of chemical fertilizers and pesticides and keep them within the international limits^55^. As Kesavan stated in their research, the use of chemical fertilizers and pesticides results in enhanced productivity over short periods, but leads to the degradation of soil health, freshwater, and biodiversity in the long term. In addition, the layout of farms in prohibited and restricted aquaculture areas should be strictly controlled, and strict total quantity control should be implemented in suitable areas. The prohibited and restricted livestock breeding areas in all districts and counties have been revised by Chongqing Environmental Protection Bureau in 2019, aiming to better manage the layout of livestock farms and reduce the risk of water environment pollution.

#### Focus on high-risk agglomeration area in nuclear density results

The high values of kernel density changed only slightly over time, indicating that the maximum scope of gathering zones showed no significant changes. The spatial position variation of kernel density at different periods is closely related to the changes of the factors in the three dimensions. The main gathering zones of medium risk levels showed some spatiotemporal differences in the three periods, but there are high-risk gathering zones in Shapingba, Yongchuan, Nanchuan, Dianjiang, Liangping and Kaizhou, and Xiushan. These phenomena are closely related with the agricultural development degree of these counties. The analysis results of such zones after further grading more clearly display the distribution of high-risk gathering zones, which, to a certain extent, reflects the distribution of medium-risk pattern spots in the vicinity of the high-risk gathering zones. These regions, such as Youting town in Dazu, Baijia town and Chengxi town in Dianjiang, the junction of Gaofeng town and Gangjia town, and Linjiang town in Kaizhou, require significant attention from the respective prevention and control departments. The cultivation system in high-risk agglomeration areas should be rationally adjusted. The crop rotation system could be adopted to restore soil fertility while controlling the amounts of chemical fertilizers and pesticides. In addition, increasing the vegetation coverage of ridges could be considered, and previous studies have proved that these measures could effectively reduce AGNPS^56^. Chongqing government has constructed river regulation works and ecological corridors in several districts and counties of the Yangtze River Basin, with the purpose of reducing soil erosion near water bodies, increasing the interception capacity of vegetation near water bodies to AGNPS, and improving the prevention and control ability of AGNPS.

#### Focus on high-value hot spots area in Getis-Ord Gi* analysis

The results of the Getis-Ord Gi* analysis clearly reflect the spatiotemporal evolution situation of high-value hot spot zones and low-value cold spot zones in the three periods in Chongqing. Over time, the dominance of high-value hot spot zones in the Midwest gradually became lower than that of the low-value cold spot zones, with a concentrated distribution in Dianjiang, Zhongxian, Fengdu, Wanzhou, Liangping, Kaizhou, Yunyang, and Fengjie. In the Midwest, the coverage areas of high-value hot spot zones and low-value cold spot zones were generally neck and neck; by contrast, in the southeast, low-value cold spot zones dominated. This trend indicates that zones with high AGNPS in northeastern Chongqing have the tendency of further gathering and diffusing, and the high-risk areas migrate toward northeastern Chongqing. Against this background, these regions deserve special attention by decision makers. Due to the considerable amount of sloping farmlands in these areas, the cultivation of such farmland should be strictly controlled^57^. In addition, soil and water conservation measures should be strengthened in these areas. In recent years, Chongqing Planning and Natural Resources Bureau has changed a large number of slope farmland into non cultivated land in order to reduce the carrying capacity of soil and water loss to pollutants, and reduce pollution risk from the source and transmission process of AGNPS.

AGNPS risk assessment is different from pollution load measurement. The accuracy of the risk results does not depend on the experimental or measured data, and may not be positively correlated with the measured data. This is similar to the multi index system assessment of NPS carried out by Huang for Taihu Lake. And according to the risk values calculated from multi-angle indicators, the risk differences in various regions are analyzed and countermeasures are proposed^35^. For example, if the I dimension value of an area is high, but the T dimension is low, that is, the measured data value is high, this does not automatically mean a high risk. The results of risk assessment are based on the reliability of the data source, factor weight and grading reference value of risk factors. It is based on the historical data to judge the trend of regional pollution risk, so as to prevent and control the risk, and this is the advantage of risk assessment in pollution prevention. Therefore, in the future, we should increase investment in data monitoring and acquisition of I, T and O dimensions and scientifically analyze the differences in the reference values of each factor in different regions.

## Conclusions

We built an ITO3dE model, analyzed the land use change during 2005–2015 in Chongqing by the combination of various spatial analysis functions including the spatiotemporal transition matrix in GIS, kernel density analysis, and Getis-Ord Gi* analysis, and clarified the influences of farmland change on AGNPS risks. The spatiotemporal variation of the pollution risk indicates that the T dimension had the highest risk level in different regions of Chongqing. This phenomenon was closely related with the widely distributed purple soil, the large differences in slope gradient and slope length, and the widely distributed sloping fields. The risk evaluation results obtained via spatiotemporal transition matrix, kernel density analysis, and Getis-Ord Gi* analysis specifically quantified the regions that require close attention in the prevention and control of AGNPS from the perspectives of risk level variation, the distribution of risk highly-concentrated zones, and the shift of high-value hot spot zones and low-value cold spot zones; we therefore provide data to support land use planning strategies as well as measures to prevent and control AGNPS.

Taken together, there would be significant influences on the Input and Output dimensions of AGNPS that promote the “one regulation, two reductions, and three basics” policies promulgated by the Chinese government, indicating areas suitable for livestock breeding in Chongqing and implementing regulatory requirements in 2018. The implementation of related policies would effectively reduce the risk levels of these two dimensions. In this study, the results suggest that there is a higher risk level in the Translate dimension. Among its influencing factors, field slope is important and can be improved by land use planning or artificial handling. Hence, related departments should focus on the farmland protection in the high-risk gathering regions, avoid farmland reclamation as far as possible, and reduce the proportion of sloping fields. At the same time, the migration of pollutants into water bodies is mainly affected by the degree of soil erodibility, so there would a positive impact that strengthening the control measures of regional soil erosion on reducing AGNPS.
